# Oxytocin Inhibition of Metastatic Colorectal Cancer by Suppressing the Expression of Fibroblast Activation Protein-α

**DOI:** 10.3389/fnins.2019.01317

**Published:** 2019-12-13

**Authors:** Mingxing Ma, Li Li, He Chen, Yong Feng

**Affiliations:** ^1^Department of Colorectal Surgery, Shengjing Hospital, China Medical University, Shenyang, China; ^2^Department of Forensic Medicine, Harbin Medical University, Harbin, China; ^3^Department of General Surgery, Affiliated Shengjing Hospital, China Medical University, Shenyang, China

**Keywords:** colorectal cancer, fibroblast activation protein-α, oxytocin, oxytocin receptor, metastasis

## Abstract

Oxytocin (OXT) and its receptor (OXTR) are present in the gastrointestinal system and are involved in gastrointestinal tumorigenesis. However, the effect of OXTR signaling on the development of colorectal cancer (CRC) and its underlying mechanisms remain unexplored. To address these issues, we first examined the expressions of OXT, OXTR, and several cancer-associated proteins using colon “tissue chips” from a spectrum of malignant progression of the colon, which included normal colon tissue, chronic colitis, colorectal adenoma, and colorectal adenocarcinoma (CAC). The results showed that the expressions of OXT and OXTR decreased gradually with the malignant progression of the disease. Stimulation of CAC tissues with OXT increased OXTR expression while down-regulated FAPα and CCL-2 protein expressions in a concentration- and time-dependent manner. Moreover, cell invasion experiment showed that OXT treatment reduced the invasion ability of colon cancer cells and blocking OXTR with atosiban blocked OXT-reduced invasion ability of human colon cancer cell lines Ls174T and SW480. The results indicate that OXT has the potential to inhibit CRC development via down-regulating the immunosuppressive proteins FAPα and CCL-2. When the OXTR signaling is weakened, colon tissues may transform to CRC. These findings also highlight the possibility of applying OXT to inhibit CRC development directly.

## Introduction

Colorectal cancer (CRC), a cancer that affects the colon, rectum, or appendix, is the third most common malignant disease in the world and the second most common cancer type in the United States (El-Shami et al., 2015), Europe, and Poland (Witold et al., 2018) in addition to being a leading cause of cancer-related mortality. With the exception of the United States, the incidence of CRC in most regions of the world is expected to increase continuously in the coming decades, due to population growth and changes in the demographic structure (Tsoi et al., 2017). Importantly, the trend toward a higher incidence of CRC primarily occurs among young adults (Austin et al., 2014), which may have a large impact on the working population and thus have a negative impact on the development of human society.

The pathogenesis of CRC involves the activation of oncogenesis and the mutation of mismatch repair genes or the inactivation of tumor suppressor genes, which further activate the oncogenic signal transduction pathways to promote proliferation, migration, invasion, and apoptosis of cancer cells (Andres et al., 2018), such as p53 (Tiwari et al., 2018). In the CRC metastases, the fibroblast activation protein-alpha (FAPα) plays a critical role. It has been reported that in all CRC samples examined, FAPα was expressed in cancer-associated fibroblasts, but not in normal colon, hyperplastic polyps, or adenoma samples. CRC cells, but not adenoma cells, can activate fibroblasts by inducing FAPα expression in order to increase their migration and invasion by releasing the transforming growth factor (TGF; Hawinkels et al., 2014). Furthermore, in mouse CRC model, cancer-associated fibroblasts with high FAPα expression induce resistance to immune checkpoint blockade by up-regulating C–C motif chemokine ligand 2 (CCL-2) secretion, recruiting myeloid cells, and decreasing T-cell activity (Chen et al., 2017). Therefore, clarifying the regulation of FAPα expression in cancer-associated fibroblasts and its associated cytokines probably provide an optional target for suppressing CRC migration.

Oxytocin (OXT) is a neuropeptide produced in both the central nervous system and peripheral tissues. OXT has been implicated in preventing the emergence of certain tumors by identifying, destroying, and eliminating mutant cells in a timely manner, such as breast cancer, prostate cancer, trophoblast-endothelial-derived tumor cells, and endometrial cancer cells (Imanieh et al., 2014; Shin et al., 2016). In the gastrointestinal (GI) tract, a lesser number of small bowel and pancreatic neuroendocrine tumors (NETs) express OXT receptor (OXTR) gene, which is significantly different from the adjacent normal tissue (Carr et al., 2013). However, the absolute expression of OXTR in the NETs varies greatly according to the types of primary tumors and is close to the somatostatin receptor type 2 in the small bowel NETs but not in the pancreatic NETs (Sherman et al., 2013). Moreover, OXT can both stimulate and inhibit the growth of certain tumors, such as osteosarcoma cells at different stages of cell differentiation (Petersson, 2008). Thus, it remains a question about the expression of OXT and OXTR in CRCs and the functions of OXTR signaling in CRC development.

To address these issues, expressions of OXT and OXTR were first identified in normal colon tissue, chronic colitis, tubular adenoma, and colonic adenocarcinoma (CAC) of tissue chips. Then, freshly excised normal colon and CAC tissues were stimulated with OXT and expression of OXTR, FAPα, TGF-β, and CCL-2 proteins were analyzed. Additionally, using Transwell migration assay, we observed effects of OXT with and without OXTR antagonist on the migration of colon cancer cells. The results highlight for the first time that OXT may inhibit CAC migration by inhibiting the expression of immune suppression-associated proteins, FAPα and CCL-2.

## Materials and Methods

In the present study, human CRC tissue chips, CRC surgical specimens, and colon cancer cell lines were used to study the effect of OXTR signaling on CRC development. The study protocols following the principles of the Declaration of Helsinki were respectively approved by the Ethics Committees of the China Medical University and the Harbin Medical University. Written informed consent was obtained from all patients.

### Tissue Collection

Tumor tissues and normal tissues adjacent to a tumor (or precancerous tissues) were collected from CRC patients. The patients were referred to either Shengjing Hospital of China Medical University or Harbin Medical University Cancer Hospital, who received conventional laparotomy between May 2016 and May 2017. The grading and staging of the CRC tumor were performed by a pathologist based on the WHO diagnostic criteria. None of the patients’ tissues received any chemotherapy or other kinds of therapies. The CRC tissues from six patients aged between 49 and 65 years (2 males and 4 females) were pathologically identified as well-differentiated CAC and were used to assess the association of OXTR signaling with CRC development. All specimens were collected and made anonymous according to ethical and legal standards.

### Detection of Cancer-Associated Proteins in the Spectrum of Human CAC Tissue Chip

The human CAC tissue chips were purchased from Alenabio (Xi’an, China), containing normal colon tissue, chronic colitis, colonic tubular adenoma, and well-differentiated CAC. The tissue chip was dewaxed using conventional methods and then incubated with hydrogen peroxide for 15 min. The first antibodies, including FAPα (1:300, Abcam), OXTR (1:300, Abcam), OXT (1:200, Abcam), and CCL-2 (1:200, Bioss) were added to the incubation overnight at 4°C. The corresponding secondary antibodies (50 μl, 1:1000) were added to the reaction that was incubated for 30 min at room temperature (21–23°C). After coloration, the photographs of the images were taken with an imaging system (Life Science), and the positive immunohistochemical staining was represented with brown stains visible under a light microscope.

### OXT Treatment and Fluorescent Immunohistochemistry of CRC-Related Proteins in CAC Tissues

Freshly dissected CAC tissue and normal colon tissue (control) were separately placed in a 5-ml centrifuge tube containing 4°C of Tyrode’s solution and allowed to acclimate for 30 min at room temperature. The tissue was cut into 1-mm square tissue pieces in a glass dish, and the same number of tissue blocks was placed in 12-well plates. In 2 ml of pre-heated 37°C Tyrode’s solution containing 0.1 nM OXT, the tissue blocks were incubated for 0, 10, 30, and 120 min, respectively. In another set of study, the tissue blocks were incubated for 30 min in the reaction containing OXT in 1 pM, 0.1 nM, and 10 nM in 2 ml of Tyrode’s solution, and then placed in liquid nitrogen for later preparation of frozen tissue sections. In the sectioning, tissues were embedded in OCT at -20°C and cut into 2-μm-thick sections.

Fluorescent immunohistochemistry was performed as previously described (You et al., 2011; Delorme and Garabedian, 2018). In brief, the cryosections of the control and CAC tissues were incubated with the following primary antibodies: FAPα, OXTR, CCL-2, and TGF-β (1:100), for 1 h after conventional permeation and blocking non-specific binding. Then, the sections were further processed with the corresponding secondary antibodies. Sections were examined with a fluorescence microscope (Nikon Eclipse FN1) through a CCD camera (Nikon DS-Ri2). To avoid false-positive or -negative immunostaining results, serial dilutions of the primary antibody, pre-absorbed primary antibody staining, and no primary and no secondary antibody controls were applied.

### Invasion Ability of Human Colon Cancer Cells

To study cell invasiveness, Transwell chambers (6.5 mm diameter, 8 μm pore size, Millipore) coated with Matrigel (BD Biosciences, Bedford, MA, United States) were used. Human colon cancer cell lines (Ls174T, ATCC, United States; SW480, Cell Bank, Chinese Academy of Sciences) of 5 × 10^5^/ml in 170 μl of serum-free medium were added to the upper chamber. The vehicle and OXT (0.1 nM) with or without atosiban (0.1 μM) were added to the upper chamber at 30 μl, respectively. Then, a medium (PRMI 1640) containing 10% fetal bovine serum was added to the lower chamber. Next, the cells could invade the Matrigel for 48 h. The non-invading cells on the upper surface of the membrane were removed with a cotton swab and the invading cells were counted in microscopic fields for each membrane under 40 × magnification.

### Data Analysis

All data are expressed as mean ± standard error of the mean. Multiple comparisons were statistically analyzed using the Mann–Whitney *U*-test or one-way analysis of variance, followed by a Student–Newman–Keuls *post hoc* test. All statistical analyses were performed using GraphPad Prism software version 5.0 and *P* < 0.05 was considered statistically significant.

## Results

### Expressions of OXT, OXTR, FAPα, and CCL-2 in the CAC Chips

**FIGURE 1:**
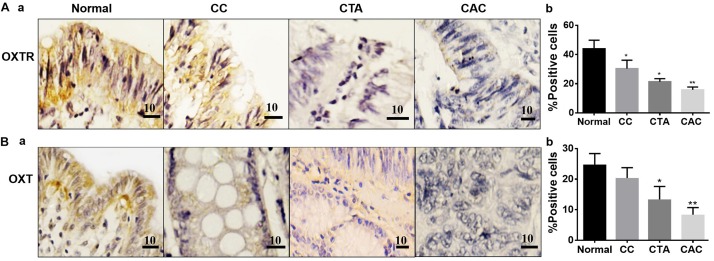
Expressions of oxytocin (OXT) receptor (OXTR) and OXT in different colon tissues. **(A)** Exemplary staining of OXTR (Aa) in normal colon tissue (Normal), chronic colitis (CC), colonic tubular adenoma (CTA), and well-differentiated adenocarcinoma of the colon (CAC) (a, from left to right) and their summaries in bar graph (b), respectively. **(B)** Exemplary staining of OXT (a) in the tissues stated in Aa and the summary (b), respectively. Positive immunohistochemical staining is indicated by brown spots. The unit of scale bars is μm. Comparisons between groups linked to the horizontal line(s) were performed by means of a one-way analysis of variance, followed by a Student–Newman–Keuls test. ^∗^*P* < 0.05, ^∗∗^*P* < 0.01, and *n* = 20–30.

To reveal the regulation of OXTR signaling in the development of CRC, expressions of OXT, OXTR, FAPα, and CCL-2 were first examined in CAC chips. The results showed that the expressions of OXT and OXTR were relatively high in normal tissue, and the expressions in chronic colitis, tubular adenoma, and well-differentiated CAC decreased gradually (Figure 1).

**FIGURE 2:**
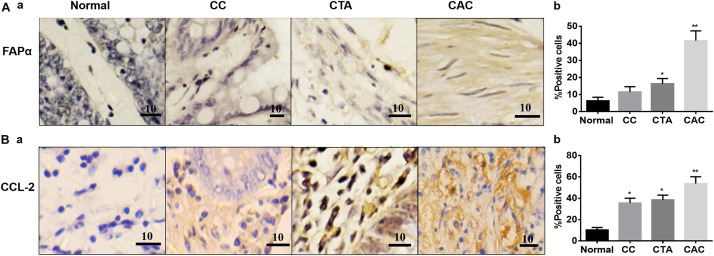
Expressions of fibroblast activation protein-α (FAPα) and C–C motif chemokine ligand 2 (CCL-2) proteins in different colon tissues. **(A)** Exemplary staining of FAPα in Normal, CC, CTA, and CAC and their summaries, respectively. **(B)** Exemplary staining of CCL-2 (a) in the tissues stated in Aa and the summary (b), respectively. Kindly refer to Figure 1 for other annotations.

In contrast, the expressions of the cancer-associated proteins FAPα and CCL-2 in the CACs were more pronounced than the normal colon tissue. In the tissue with chronic colitis, CCL-2 but not FAPα was also significantly high compared to the control (Figure 2). These findings are consistent with other reports in cancerous tissues (Henriksson et al., 2011; Chen et al., 2017).

### Time- and Dose-Associated Effects of OXT on the Expression of Different CRC Molecules

Similar to the findings on the chips, OXT and OXTR were also observed in patients’ colon tissues. In normal tissues adjacent to the CAC, both the OXT and OXTR were observed in the myenteric neural plexus and submucosal tissues. In the CAC, OXT was mostly absent while the OXTR was weak (Supplementary Figure 1).

**FIGURE 3:**
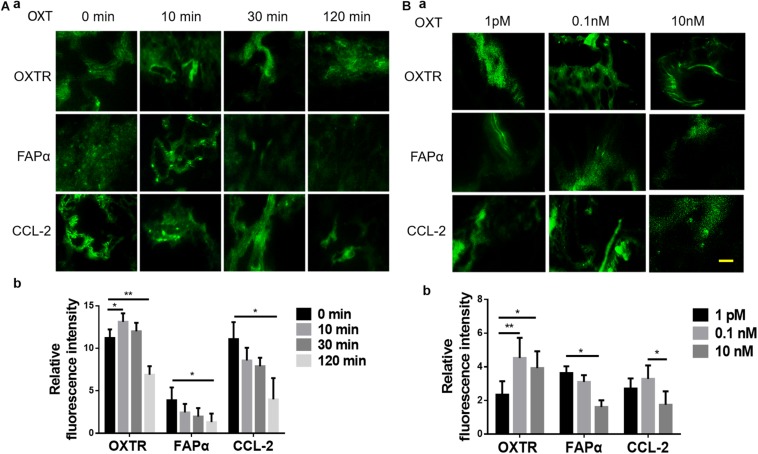
Time- and dose-associated effects of OXT on the expression of OXTR, FAPα, and CCL-2 in fresh human colon tissues of patients with colorectal cancer (CRC). **(A,B)** Time-associated effect and the dose-associated effect in exemplary fluorescent images (a) and the summaries in bar graphs (b) for FAPα **(A)** and CCL-2 **(B)**, respectively. The scale bars are equal to 10 μm. Kindly refer to Figure 1 for other annotations.

The negative association between OXT/OXTR and FAPα suggests the presence of a causal relationship between a decrease in OXTR signaling and the development of CRC. Since the biological effect of OXT possesses significant features of time and dose dependence, as shown in OXT neurons (Wang and Hatton, 2006; Wang et al., 2006), we first examined the temporal effect of OXT on the expression of OXTR, FAPα, and CCL-2. The results showed that in normal tissues, OXT increased the expression of OXTR at 10 and 30 min; this effect decreased significantly after 120 min; FAPα and CCL-2 proteins decreased significantly after 10 min (Figure 3A). Furthermore, in response to increased concentrations of OXT (1 pM, 0.1 nM, 10 nM), OXTR levels increased significantly, but FAPα levels decreased significantly. CCL-2 increased significantly with 0.1 nM OXT, but decreased significantly with 10 nM (Figure 3B). This finding supports presence of a physiological action of OXT in colon tissues.

**FIGURE 4:**
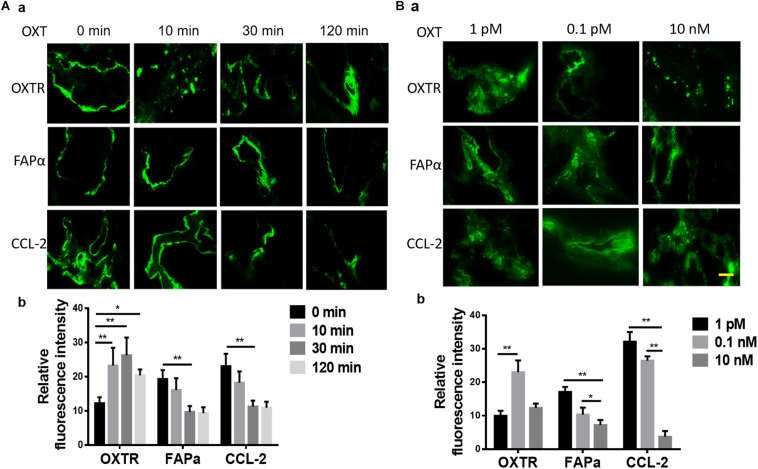
Time- and dose-associated effects of OXT on the expression of OXTR, FAPα, and CCL-2 in CAC tissues of patients with CRC. **(A,B)** Time-associated effect and the dose-associated effect in exemplary fluorescent images (a) and the summaries in bar graphs (b) for FAPα **(A)** and CCL-2 **(B)**, respectively. The scale bars are equal to 10 μm. Kindly refer to Figure 1 for other annotations.

In CAC, treatment with OXT for 10 min or 30 min significantly increased the expression of OXTR but decreased FAPα and CCL-2; OXT-increased expression of OXTR became insignificant at 120 min when FAPα and CCL-2 did not show further decrease (Figure 4A). This finding is consistent with the general regulation of receptor internalization and decomposition after prolonged hormonal stimulation.

Further analysis of the response of CAC-associated tissues to different doses (1 pM, 0.1 nM, or 10 nM) of OXT stimulation for 10 min revealed that OXT dose-dependently altered the expression of OXTR, FAPα, and CCL-2 in CACs. As shown in Figure 4B, compared to 1 pM OXT, 0.1 nM OXT markedly reduced the expression of FAPα and CCL-2 while increasing OXTR expression. A higher dose of OXT (10 nM) further reduced FAPα and CCL-2, but its effect on OXTR expression did not significantly differ from the effect of 1 pM OXT. These time- and dose-dependent OXT effects are in agreement with OXT effects in neural tissues (Wang and Hatton, 2006; Wang et al., 2006).

Noticeably, OXT did not significantly affect the expression of TGF-β at different times and doses (Supplementary Figure 2).

### Effects of OXT on the Invasion Ability of Human Colon Cancer Cells

**FIGURE 5:**
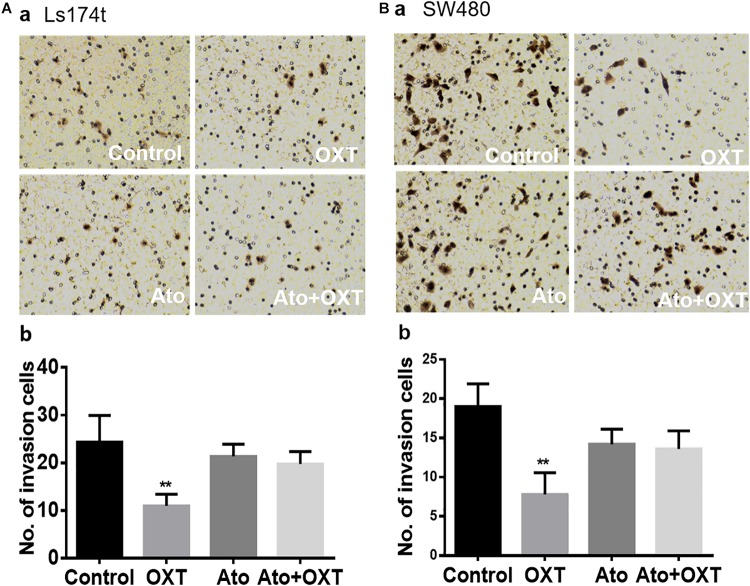
Effects of OXT on the Transwell ability of CAC cells. **(A,B)** Representative images of Transwell membranes for the Ls174T (**A**a) and SW480 (**B**a) colon cancer cell lines and their summaries (**A**b,**B**b), respectively. The four panels as marked in white represent the effects of the vehicle, OXT, atosiban, and atosiban plus OXT, respectively. ^∗∗^*P* < 0.01 compared to vehicles.

To establish a causal relationship between OXTR signaling and CRC cell metastases, the study evaluated the role of OXTR signaling in the invasion ability of human colon cancer cells using the Matrigel invasion/Transwell migration assay, with six duplicates in each group. As shown in Figure 5, OXT (0.1 nM) significantly reduced the Transwell number of colon cancer cell line, Ls174t (Figure 5A) and SW480 cells (Figure 5B). By contrast, pretreatment with OXTR antagonist atosiban (0.1 μM) did not have a significant effect on Transwell activity by itself; however, it did block the inhibitory effects of OXT on this Transwell activity. This finding is in line with the inhibitory effect of OXT on the expressions of FAPα and CCL-2.

## Discussion

The present study found that OXT and OXTR not only are present in colon tissues but also correlate with CAC migration. Clearly, the expressions of OXT and OXTR in CAC tissues were low, whereas activation of OXTR signaling reduced the expression of FAPα and CCL-2. Importantly, blocking OXTR activation inhibited OXT-induced reduction of colon cancer cell migration in both types of colon cancer cell lines. These findings highlight the possibility of suppressing CRC metastases by applying OXT directly.

### General Anticancer Effect of OXT and Its Approaches

Oxytocin is a neuropeptide composed of nine amino acids, which is widely distributed throughout the body and plays an important role in various bodily functions (Yang et al., 2013). In addition to promoting breastfeeding and childbirth (Hatton and Wang, 2008), OXT can directly or indirectly inhibit tumorigenesis. For example, chronic exposure to social stress is common in humans, especially in nursing mothers (Murgatroyd et al., 2015) and can increase the risk of inflammation-related CRC (Peters et al., 2012; Antoni and Dhabhar, 2019). Intranasal application of OXT reduces maternal depression to relieve social stress and pulsatile pattern of OXT actions can directly suppress precancerous lesions of the mammary glands. The later effect is associated with suppression of oxidative stress-induced expressions of phosphorylated extracellular signal-regulated protein kinase 1/2 and cyclooxygenase-2, as shown in rats (Liu et al., 2016).

This anticancer effect is also associated with the immune regulatory functions of OXT. Intranasal OXT application to F1 rat dams was reported to increase serum interferon-γ level in F2 juvenile female offspring (Murgatroyd et al., 2016), which is associated with apoptosis of small-cell lung cancer (Zhou et al., 2006). It was also reported that peripheral use of OXT reduced stress-induced visceral hypersensitivity and activation of enteric glial reactivity (Xu et al., 2018), thereby increasing the defense of GI tract to inflammatory challenges. In the present study, we also found that OXT can reduce the expression of CCL-2, which helps to restore immune checkpoint blockade and increase T-cell activity to inhibit the development of CRCs (Chen et al., 2017). Nevertheless, there is still a need to collect direct evidence of the suppression effect of OXT from CRC *in vivo*; OXT can suppress carcinogenesis at multiple levels including the colon tissue as discussed below.

### Protective Effects of OXT and OXTR Signaling in Colon Tissue

In association with the body’s immune functions, OXT has been recognized to maintain immune surveillance, defense, and homeostasis through many approaches (Li et al., 2017). The insufficient OXT and dysfunctions of OXTR signaling are likely associated with varieties of immune lesions, including carcinogenesis (Hou et al., 2016; Adamo et al., 2018). Our present findings further support this view by presenting the reduced expressions of OXT and OXTR in CAC tissues and by the suppressive effects of exogenous OXT on the expression of CRC metastasis-associated proteins, FAPα and CCL-2.

Colon OXT-secreting cells can influence CRC development through autocrine and paracrine functions. OXT and OXTR are widely expressed in human GI tract (Monstein et al., 2004; Ohlsson et al., 2006). They are mainly present in the nerve cell bodies and in the nerve fibers of the myenteric and submucosal ganglia (Ohlsson et al., 2006) in the jejunum, ileum, proximal colon, and distal colon (Yu et al., 2011). In addition to enteric neurons, the proximal colonic muscle strips (Wang et al., 2016) and crypt-villus enterocytes (Klein et al., 2013; Welch et al., 2014) also express OXTRs.

Unlike the inhibitory effect of the brain OXT on the feeding and mobility of the GI tract (Sabatier et al., 2013), direct effect of OXT on the GI tract is to increase its motility by causing smooth muscle contraction, as shown in the stomach (Qin et al., 2009) and the duodenum (Li et al., 2007). Thus, reduced OXT and OXTR signaling in the colon tissues likely reduce the movement of colon, which creates a favorable microenvironment for the accumulation of inflammatory agents and carcinogens, thereby facilitating CRC development.

Consistently, OXT can reduce necrotizing enterocolitis, a GI inflammatory disease of unknown etiology (Gross Margolis et al., 2017). In OXTR knockout mice, the intestinal villi and crypts were shorter, intestinal permeability to macromolecules was greater, and experimental colitis was more severe than wild-type mice (Welch et al., 2014). In the present study, we further identified that OXT-induced reduction of CCL-2 is also a manifestation of the anti-inflammatory effect of OXT in the GI system. Thus, the disruption of OXTR signaling in the GI tract can impair the immune defense function of OXT to carcinogenesis under the influence of oncogenic genetic and environmental factors.

### Inhibitory Effects of OXT on CRC Migration

Oxytocin is involved in the inhibition of metastasis of many types of cancers. For example, OXT inhibits metastatic ovarian cancer by suppressing the matrix metallopeptidase-2 expression and vascular endothelial growth factor (Ji et al., 2018). OXT down-regulates the invasion of head and neck squamous cell carcinoma cells by up-regulation of early growth response-1 and the subsequent increase in p53, and phosphatase and tensin, and p21 expression (Kim et al., 2017). The present Matrigel invasion study also confirms that OXT significantly inhibits the migration of colon cancer cells, which is mediated by OXTR. They together strongly support the inhibitory role of OXTR signaling in metastatic cancers, including CAC.

Migration involves many signaling pathways, such as integrin αvβ6 (Peng et al., 2018), TGF-β (Tiwari et al., 2018), and guanylate cyclase C (Rappaport and Waldman, 2018) as well as FAPα and CCL-2. In the present study, we specifically investigated the participation of FAPα and its associated proteins. Clearly, FAPα is a target for OXT suppression of CAC cell migration, since OXT treatment significantly reduced the expression of FAPα. Clearly, both CCL-2 and TGF-β are the downstream signals of FAPα (Henriksson et al., 2011; Meulendijks et al., 2016; Chen et al., 2017; Mohr et al., 2017), and OXT decreased CCL-2 expression but not TGF-β. Thus, the major downstream signal of OXTR-FAPα signaling is CCL-2 but not TGF-β. However, TGF-β could function as an upstream signal of the FAPα to influence CRC development independently. Noticeably, OXT suppression of CRC migration through the FAPα-CCL-2 signaling is dominant over the migration-promoting effect of TGF-β signaling as proved by our migration study. This finding is consistent with the anti-inflammatory effect of OXT and its possible function in curbing the migration of CAC cells, as previously studied (Wang, 2016). Thus, our study highlights the therapeutic value of suppression of CRC migration, at least for CAC.

## Conclusion

**FIGURE 6:**
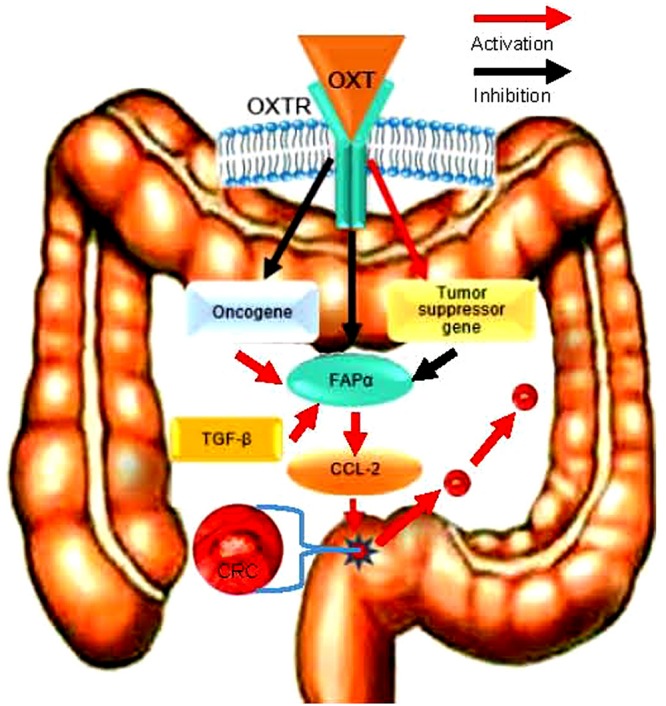
Schematic diagram of the pathway for OXT inhibition of CRC metastasis. FAPα activity is under the facilitatory influence of TGF-β and the inhibitory influence of OXT; increased OXTR signaling can antagonize the effect of TGF-β and cause the inhibition of CCL-2 action, thereby suppressing FAPα-associated CRC cell migration/metastasis.

Colorectal cancer development is associated with the reduction of OXTR signaling since OXT can suppress FAPα and CCL-2 expressions and their associated CAC migration via OXTR (Figure 6). Since OXT is a safe agent in clinical application and that the CRC is readily accessible than other GI cancers, it is possible to suppress CRC metastasis by direct application of OXT.

It is worth noting that our observations are limited to a well-differentiated CAC, and we cannot rule out the possibility of different expression patterns of OXT and OXTRs, as well as their role in other types of human CRC. In addition, prior to conducting clinical trials of OXT treatment for CRCs, further observation of the anti-metastatic effects of OXT is required, particularly by *in vivo* approaches.

## Data Availability Statement

All datasets generated for this study are included in the article/Supplementary Material.

## Ethics Statement

The study was approved by Shengjing Hospital of China Medical University. Ethical Approval Letter of Scientific Research (Chinese Original Translation). Ethical Document No. 2016PS255K. Research Project: Studies on the expressions of oxytocin and its receptor in colorectal cancer. Department of application: Department of Colorectal Surgery. Principal Investigator: Mingxing Ma. Contents Reviewed (1) Designation: Patient Consent Form and (2) Eligibility of the Researcher: Approaches of Sampling and others. Conclusion of Review: This project meets the requirements of ethical principles and performing this project is granted. Granter: Ethical Committee of China Medical University (Official Seal). Date of Approval: May 31, 2016. Valid for 4 years from the approval date.

## Author Contributions

MM and LL collected and analyzed the data. MM and HC wrote the first draft of the manuscript. HC and YF conceived the project.

## Conflict of Interest

The authors declare that the research was conducted in the absence of any commercial or financial relationships that could be construed as a potential conflict of interest.
