# Supplementary material for: Oxytocin Inhibition of Metastatic Colorectal Cancer by Suppressing the Expression of Fibroblast Activation Protein-α
Source: Front Neurosci. 2019 Dec 13;13:1317. doi: 10.3389/fnins.2019.01317 (PMC6923180; doi:10.3389/fnins.2019.01317)
Supplement: Supplementary file 1 [file Data_Sheet_1.pdf]

Supplementary Material

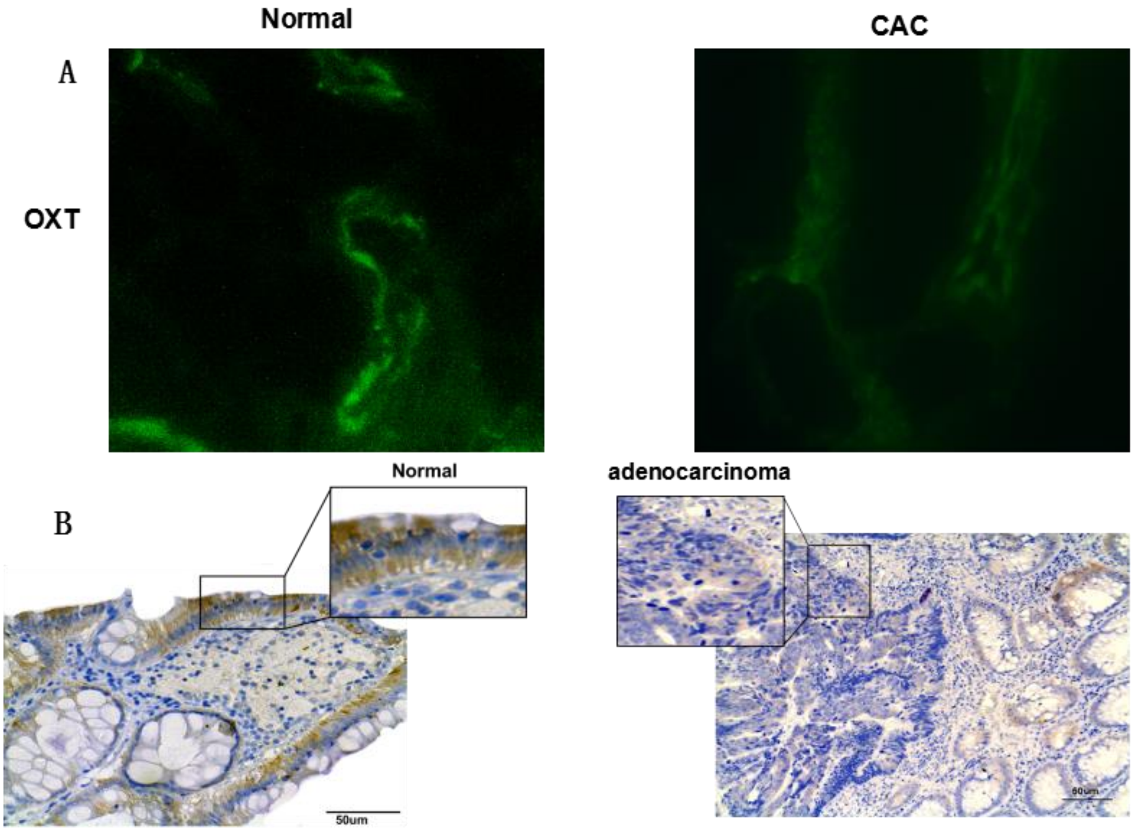

Supplementary Figure 1. OXT expression in normal and CAC tissues of CRC patients. (A) Normal colon tissues in fluorescent immunohistochemistry (a, in green) and in H&E staining (b, in brown); (B) CAC tissues in fluorescence microscopy (a) and in H&E staining.

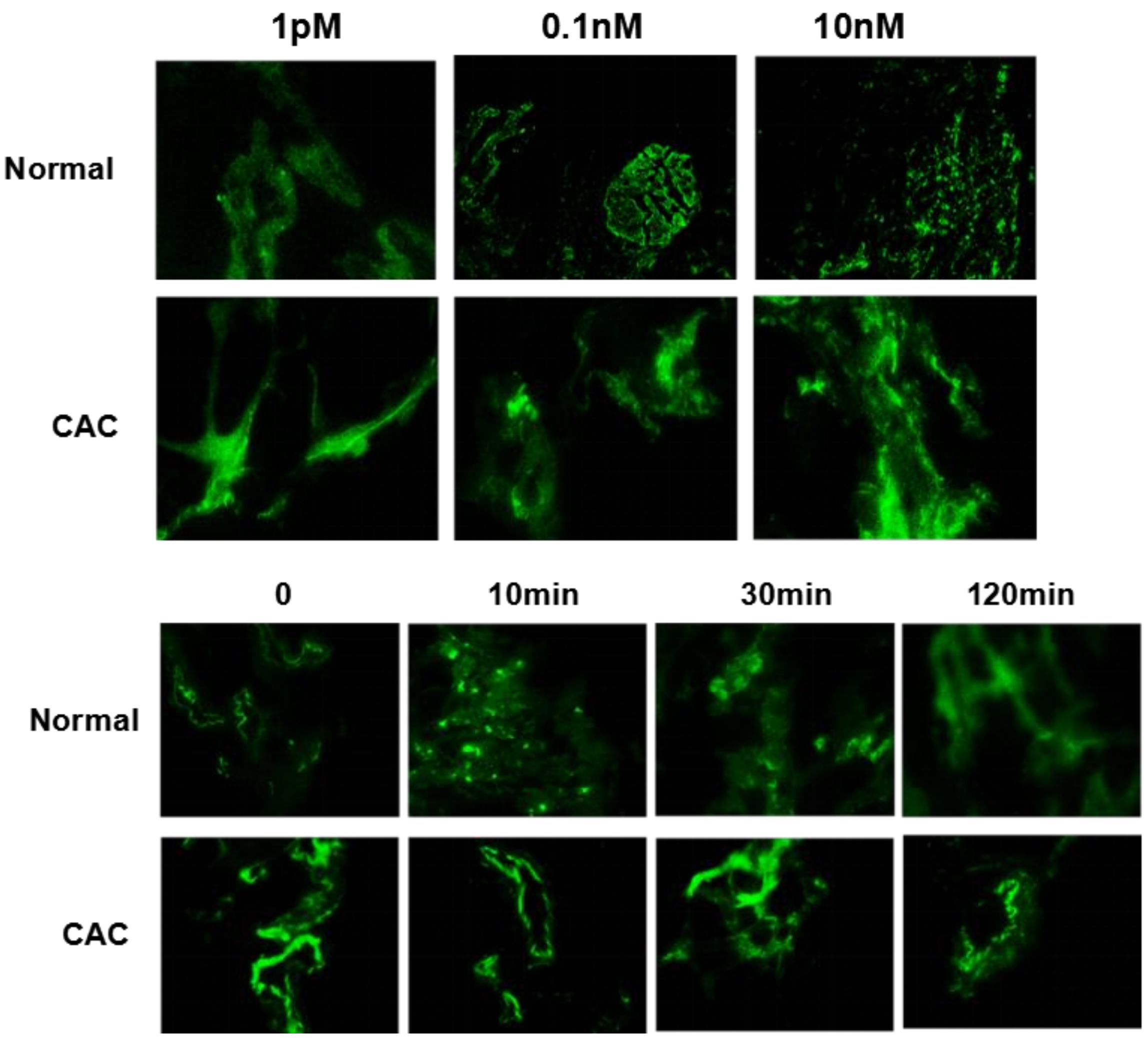

Supplementary Figure 2. Effects of OXT on the expression of TGF-β in CAC tissues of patients with CRC.
